# Chemical Characterization and Aquatic Toxicity of Firefighting Runoff—Linking Organic Profiling to Multi-Trophic Bioassays in the One Health Framework

**DOI:** 10.3390/molecules31101554

**Published:** 2026-05-07

**Authors:** Alicja Trawińska, Maciej Tankiewicz, Kamil Pająk, Monika Cieszyńska-Semenowicz, Andrzej R. Reindl

**Affiliations:** Department of Environmental Toxicology, Faculty of Health Sciences, Medical University of Gdansk, M. Sklodowskiej-Curie 3a Str., 80-210 Gdansk, Poland; alicja.trawinska@gumed.edu.pl (A.T.); maciej.tankiewicz@gumed.edu.pl (M.T.); cieszynskam@gumed.edu.pl (M.C.-S.); andrzej.reindl@gumed.edu.pl (A.R.R.)

**Keywords:** firefighting runoff, industrial fires, multi-trophic bioassays, GC-MS/MS, chemical fingerprinting, public health, One Health

## Abstract

This study investigates the organic chemical content and ecological impact of firefighting runoff collected from real-world fire scenarios. To establish a direct link between chemical composition and environmental hazard, a comprehensive analytical framework was employed, integrating molecular fingerprinting via gas chromatography–tandem mass spectrometry (GC-MS/MS) with a multi-trophic battery of bioassays, including *Aliivibrio fischeri*, *Heterocypris incongruens*, and *Sinapis alba L*. The chemical characterization revealed highly heterogeneous profiles dominated by esters (up to 41%), alcohols (up to 25%), and phenols (up to 22%). A unique molecular marker, nitriles (15.9%), was identified in tire-related fire effluents, which corresponded with potent metabolic suppression in the Toxi-ChromoTest™. Ecotoxicological results demonstrated that most effluents reached Class IV (high acute toxicity), with universal 100% lethality observed in samples from large-scale incidents. Furthermore, a significant stimulatory effect was detected in *S. alba* (growth stimulation up to 12%) for scenarios involving polyurethane foam, illustrating the selective toxicity of specific molecular groups. Beyond ecological degradation, the high phenolic and nitrile loads identified across multiple scenarios represent a substantial public health risk, as these persistent contaminants can infiltrate groundwater, bypass conventional water treatment, and bioaccumulate in the human food chain. The findings suggest that the synergistic effect of hydrophobic xenobiotics and firefighting foams poses a severe threat to both aquatic biodiversity and human chemical safety. This research emphasizes that linking molecular fingerprinting with multi-level bioindicators is essential for a holistic risk assessment of firefighting operations.

## 1. Introduction

Water is a fundamental component of the global ecosystem, essential for human life, economic stability, and the maintenance of biodiversity. However, the quality of surface and groundwater is increasingly compromised by anthropogenic activities. Among these, sudden and high-intensity events such as industrial and urban fires represent a significant, yet often under-evaluated, source of chemical contamination. The chemical complexity of firefighting runoff is not merely a sum of the initial materials but a result of dynamic thermolysis, pyrolysis, and de novo synthesis occurring under extreme thermal conditions.

During combustion, in addition to heat and smoke with ash and soot (particulate matter), a wide array of toxic substances is released into the air, soil, and water, including polycyclic aromatic hydrocarbons (PAHs), volatile and semi-volatile organic compounds (VOCs, SVOCs), heavy metals, dioxins and furans (PCDD/Fs), and toxic gases such as carbon monoxide, hydrogen cyanide, and sulfur dioxide [1,2,3,4,5,6,7]. What is more, during the fire, compounds included in the extinguishing agents add to the total chemical load of compounds, forming a heterogeneous mixture exhibiting various physicochemical properties. Specifically, the use of Aqueous Film-Forming Foams (AFFFs) introduces fluorinated surfactants that significantly alter the surface tension of the runoff, facilitating the transport of hydrophobic pollutants.

The environmental impact of these incidents is inextricably linked to human and animal health through the “One Health” paradigm, which recognizes that the human health is closely attributed to the health of animals and our co-shared environment. Recent research indicates that the chemical contamination of aquatic ecosystems resulting from firefighting activities may pose a severe threat [8,9,10,11]. Many substances released during such incidents are highly toxic to aquatic life, exhibiting both acute and chronic toxicity while persisting in the environment [12]. Many substances released during such occurrences, particularly PAHs and PFAS (per- and polyfluoroalkyl substances), are classified by the International Agency for Research on Cancer (IARC) as Group 1 carcinogens, contributing to a 14% increased risk of cancer-related mortality among firefighters. The molecular stability of PFAS, commonly referred to as “forever chemicals,” ensures their exceptional persistence in the environment. Improper management of firefighting wastewater can lead to long-term degradation, with release of contaminants like dioxins and heavy metals persisting for over 15 years [8], eventually entering the human food chain through bioaccumulation and biomagnification in agricultural crops and groundwater.

This highlights a critical research gap, as systematic studies on the ecotoxicity of runoff from real-world industrial and warehouse fires remain scarce. Most available data are derived from small-scale laboratory models or forest fires [3,13,14,15], failing to represent the complex chemical mixtures generated at real-life disaster sites. For instance, emissions from uncontrolled tire fires have been estimated to exhibit mutagenic activity levels up to 13,000 times higher than those from coal combustion, posing an acute risk to nearby communities through respiratory distress and potential neurological damage [16,17,18]. Furthermore, the kinetics of pollutant mobilization strongly depend on the pH of the firewater, which can range from highly acidic to strongly alkaline, depending on the stored materials and extinguishing agents used.

Assessing the actual ecological and human risk requires more than chemical analysis alone. Relying solely on chemical detection may overestimate hazards, as the bioavailability of chemicals, such as metals under alkaline conditions, significantly influences their actual toxic effects [12]. For example, the speciation of chromium (Cr) between its trivalent and hexavalent forms determines both its mobility in the water column and its carcinogenic potential. Bioindicators support the actual hazard assessment by reflecting the biological responses to bioavailable contaminants. They serve as vital tools for quantifying environmental impact of chemicals, providing a direct measure of biological stress [19,20]. Biotests determine the concentration levels at which organisms can survive and function [21]. In addition to standard test batteries like Daphtoxkit F (crustaceans), Rotoxkit F (rotifers), and Spirodela toxkit (macrophytes) [3,22], more specialized tools such as the Ostracodtoxkit F (direct sediment contact) and the Toxi-ChromoTest (enzyme inhibition) provide high-resolution data on metabolic and chronic effects of compounds.

The aim of this study was to address the lack of empirical evidence regarding the environmental and health-related impacts of real-life fires. The aim of this study was to conduct a comprehensive ecotoxicological assessment of firefighting runoff collected from 10 real fire incidents, representing a broad spectrum of fuels and industrial categories, including a tire landfill, municipal waste collection points, commercial retail stores, large storage warehouses, automotive workshops, and facilities containing polyurethane (PUR) foam. In addition to the ecotoxicological tests battery, advanced screening analyzes using GC-MS/MS technique were performed to identify the specific groups of chemical compounds present in the firefighting runoff. This enabled a detailed molecular characterization of pollutants profiling, bridging the gap between chemical composition and biological impact. By combining a battery of biotests spanning multiple trophic levels with chemical fingerprinting, this study provides the first systematic cross-sectional analysis of how different real-world fire scenarios threaten the aquatic environment.

## 2. Results

### 2.1. Bioluminescence Inhibition Measurement in Aliivibrio fischeri (Microtox^®^ Assay)

**Figure 1:**
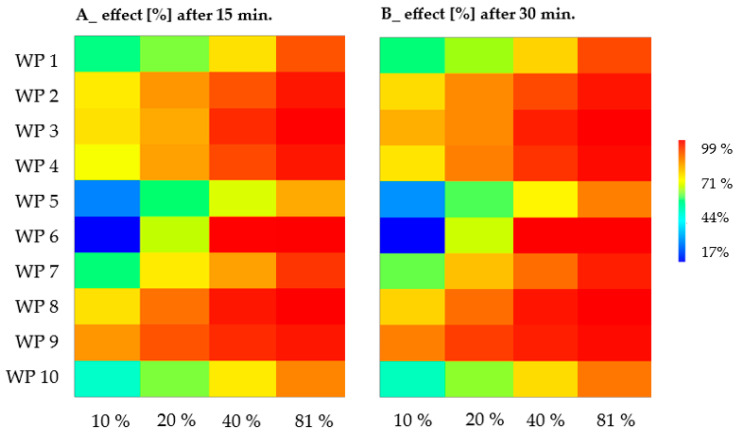
Percentage of bioluminescence inhibition in *Aliivibrio fischeri* after 15 (**A**) and 30 (**B**) minutes of incubation with firefighting runoff samples (WP1–WP10).

The Microtox^®^ 81.9% Basic Test protocol revealed pronounced differences in toxicity towards luminescent bacteria among the tested samples, as summarized in Table 1. The inhibition of luminescence in *Aliivibrio fischeri* increased proportionally with both exposure time and sample concentration (Figure 1), indicating potent acute toxic effects in several instances. Toxicity classes were assigned according to the methodology of Persoone et al. [23], utilizing the most sensitive endpoint—the EC_50_ value after 15 and 30 min of exposure.

For highly toxic samples (WP2, WP3, WP6, WP9, and WP10), the initial concentration range resulted in near-total inhibition of bacterial luminescence, precluding reliable EC_50_ determination under standard assay conditions. To address this, these samples were pre-diluted prior to testing (two-fold for WP2 and WP6; five-fold for WP10; and nine-fold for WP9). This approach allowed for the determination of measurable EC_50_ values, which were subsequently recalculated to the original sample concentrations. In contrast, the remaining samples exhibited measurable toxicity within the standard range, enabling a direct and precise quantitative comparison.

Based on the toxicity units (TUs) derived from EC_50_ values, the effluents were classified into various categories ranging from slightly to extremely toxic. In most cases, toxicity levels increased with prolonged exposure.

### 2.2. Bacterial Enzyme De Novo Synthesis Inhibition (Toxi-ChromoTest™)

**Figure 2:**
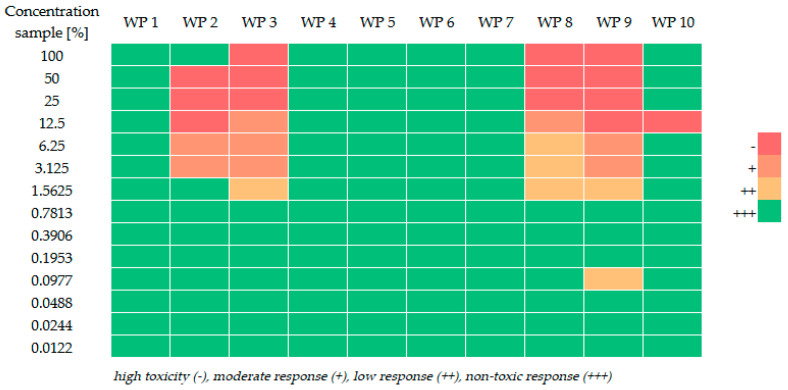
Qualitative assessment of bacterial de novo enzyme synthesis inhibition using the Toxi-ChromoTest™ for firefighting runoff samples (WP1–WP10).

Distinct variations in color intensity were observed both between different fire scenarios and across the dilution series (see Figure 2). Samples WP3, WP8, and WP9 exhibited the most potent toxic effects at high concentrations, characterized by a total absence of chromogenic reaction (no color development). As these samples were progressively diluted, a gradual increase in blue color intensity was noted, signaling a transition toward non-toxic levels. However, the dose–response relationships were not strictly monotonic in all cases. Certain samples displayed non-linear patterns, where moderate or low responses occurred at intermediate dilutions, while non-toxic results were recorded at both higher and lower concentration levels.

At the maximum tested concentration (100%), samples WP3, WP8, and WP9 showed no visible enzyme activity. Conversely, samples WP1, WP4, WP5, WP6, WP7, and WP10 exhibited non-toxic responses across a broad concentration range, including the undiluted state. With increasing dilution, all samples eventually reached a color intensity comparable to the negative control. The specific concentration thresholds required to reach a non-toxic state varied significantly.

### 2.3. Phytotoxicity Assessment Using Sinapis alba L.

The results, summarized in Table 2, revealed a highly heterogeneous phytotoxic response, reflecting the diverse chemical compositions of the 10 fire scenarios.

The analysis of seed germination provided a preliminary threshold for phytotoxicity. A critical finding was the complete suppression of germination (100% inhibition) in three specific samples (WP3, WP8, and WP9). In contrast, most other samples exhibited germination rates comparable to the control, with the notable exception of sample WP2, where germination was nearly abolished (only two seeds germinated).

Root growth inhibition, measured for both mean and longest root length, proved to be a significantly more sensitive endpoint than germination by capturing a broader spectrum of physiological stress across the tested samples. The magnitude of this inhibition ranged from complete stunting to notable growth stimulation, with samples WP2, WP3, WP8, and WP9 being categorized into the highest toxicity class (Class IV) according to Persoone et al. [23].

Interestingly, samples WP5 and WP6 demonstrated negative inhibition values reaching as low as 12.25%, signaling a stimulatory effect on root development; this biphasic response, or hormesis, may be attributed to the presence of diluted nitrogenous compounds or other nutrients in the runoff that act as fertilizers rather than toxins at lower concentration levels.

### 2.4. Toxicity to Aquatic Invertebrates—Ostracodtoxkit F™

**Figure 3:**
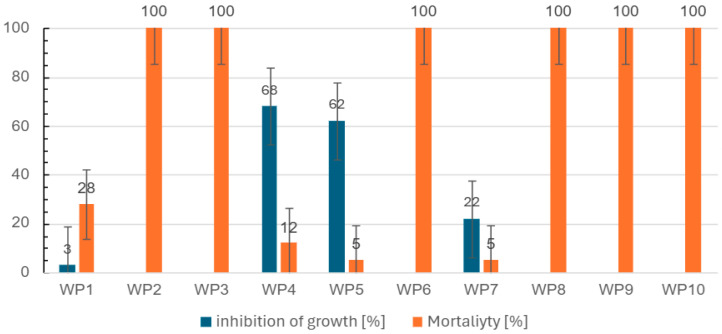
Response of freshwater ostracod *Heterocypris incongruens* exposed to firefighting runoff samples (WP1–WP10).

The Ostracodtoxkit F™ bioassay revealed a profound toxic impact of several firefighting runoff samples on aquatic invertebrates (see Figure 3). Both mortality and growth inhibition of the ostracods varied significantly across the ten scenarios, reflecting a broad spectrum of toxic potency. In accordance with the methodology of Persoone et al. [23], toxicity classes were assigned based on the most sensitive endpoint recorded for each sample.

The total mortality (100%) of the test organisms observed in most of the samples, specifically in WP2, WP3, WP6, WP8, WP9, and WP10, demonstrated severe acute toxicity. Conversely, other samples exhibited partial mortality accompanied by measurable growth inhibition, indicating significant sublethal toxic effects. For the samples where organisms survived, growth inhibition proved to be a highly sensitive parameter for assessing the physiological stress caused by the chemical constituents of the runoff.

Based on the most sensitive endpoints, the investigated samples were categorized into Persoone’s toxicity classes, ranging from slightly toxic (Class II for WP1 and WP7) to highly or extremely toxic (Class IV for WP2, WP3, WP4, WP5, WP6, WP8, WP9, and WP10).

### 2.5. Chemical Screening and Functional Group Profiling by GC-MS/MS

**Figure 4:**
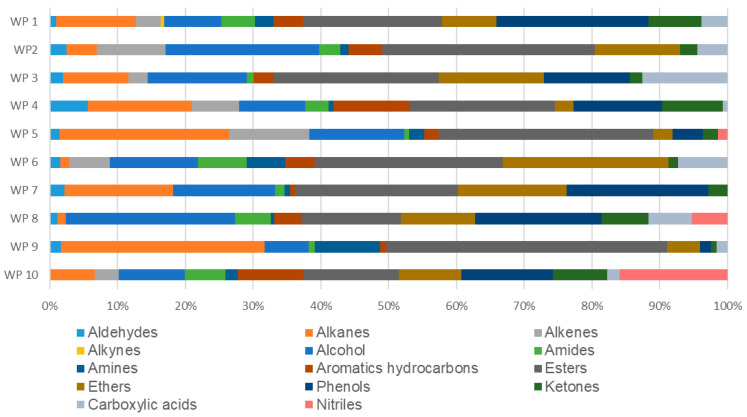
Qualitative distribution of organic functional groups in firefighting runoff samples (WP1–WP10) identified by GC-MS/MS. Data are expressed as the percentage area of identified chromatographic peaks for each chemical class. Functional groups include aldehydes, alkanes, alkenes, alkynes, alcohols, amides, amines, aromatics, esters, ethers, phenols, ketones, carboxylic acids, and nitrile.

The qualitative screening performed via GC-MS/MS allowed for the identification and categorization of organic constituents in the firefighting runoff into major functional organic groups (Figure 4). The chemical landscape of the samples was dominated by esters, alcohols, and phenols, although their relative abundance varied significantly across the ten fire scenarios (WP1–WP10), explaining the heterogeneous ecotoxicological responses observed in the bioassays. They originate from pyrolysis and incomplete combustion of common materials such as plastics, wood, textiles and synthetic polymers. These compounds arise specifically from thermal breakdown of polymers (e.g., polyesters releasing esters, cellulose yielding alcohols) and lignin in wood producing phenols, with firefighting water extracting the resulting volatile and semi-volatile products from ash, smoke, and residual matter. Their predominance directly reflects the high reactivity of the fire process, where intense oxidation and pyrolysis under oxygen-limited conditions—coupled with temperatures exceeding 500 °C—favor secondary product synthesis over complete combustion to CO_2_ and H_2_O, establishing these compounds as biomarkers of incomplete combustion [24,25].

Esters were the most prevalent group in several scenarios, reaching a maximum of 41% in WP9, 32% in WP5, and 31% in WP2. The high concentration of esters, often associated with plasticizers and synthetic materials, likely contributed to the overall chemical load and potential sublethal effects. Alcohols also represented a substantial fraction, particularly in WP2 (22%) and WP8 (25%), where they may have enhanced the solubility and bioavailability of other more toxic hydrophobic compounds.

Phenols and aromatic compounds, known for their high toxicity and persistence, were notably abundant in specific samples. Samples WP1, WP7 and WP8 exhibited high phenol contents (22%, 21% and 19%, respectively), which correlates with their classified toxicity. Furthermore, aromatic compounds showed a peak in WP4 (11%), WP9 (10%) and WP10 (9.76%), scenarios likely involving the combustion of complex polymers and industrial chemicals. Interestingly, nitrile compounds were uniquely prominent in WP10 (16%) and WP8 (5.2%), serving as chemical markers for the nitrogen-containing materials or specific extinguishing agents used in those incidents.

Alkanes and alkenes, representing products of incomplete combustion and fuel residues, were found in high proportions in WP9 (30% alkanes) and WP5 (25% alkanes). While these compounds often exhibit lower acute toxicity than aromatics, their presence in such high relative abundance significantly influences the physicochemical properties of the runoff, such as its lipophilicity. The diverse presence of nitrogen-containing groups, including amides (up to 7.2% in WP6) and amines (up to 9.7% in WP9), further complicates the toxicological profile of the effluents, potentially leading to the synergistic effects observed in the Microtox^®^ and Toxi-ChromoTest™ assays.

## 3. Discussion

The intersection of fire events and the subsequent release of highly contaminated fire-extinguishing water represent one of the most complex vectors for acute and chronic environmental pollution. The analytical results derived from GC-MS/MS in this study provide a critical foundation for understanding the chemical etiology of firewater runoff toxicity. Profiling these effluents revealed an extremely dense matrix of organic contaminants, including polycyclic aromatic hydrocarbons (PAHs, such as anthracene, phenanthrene, fluoranthene and pyrene), unburned fuel hydrocarbons (BTEX), chlorinated dioxins, phenols (e.g., benzenol, cresol and eugenol) and highly reactive intermediate combustion products, which is consistent with the foundational work of Noiton, D., Fowles, J., & Davies, H. [12]. When these chemical signatures are rigorously mapped against the multi-trophic ecotoxicological responses observed in the studied battery of bioassays, it becomes unequivocally evident that traditional, single-species monotonic risk assessments underestimate the ecological peril posed by firewater runoff.

One of the most persistent scientific challenges in evaluating the ecotoxicity of such runoff is the profound deviation between the predicted toxicity of individual chemical constituents and the observed reality of the complex environmental mixture. This phenomenon, frequently termed the “toxic cocktail” effect, results in synergistic and supra-additive toxicity, where the combined biological impact of the effluents significantly exceeds the simple arithmetic summation of the individual components’ toxicities [26]. Our results demonstrate this clearly in samples such as WP2 and WP8, which contained substantial quantities of alcohols (22.3% and 25%, respectively). As Sato and Nakajima [27] established, high concentrations of completely miscible alcohols such as ethanol or isopropyl alcohol significantly reduce the overall polarity and dielectric constant of the bulk aqueous phase. This shift in the physicochemical properties of the matrix likely acted as an anti-hydrophobic co-solvent, facilitating the mobilization and increasing the bioavailability of highly toxic, hydrophobic xenobiotics that would otherwise remain sequestered in the particulate phase.

**Figure 5:**
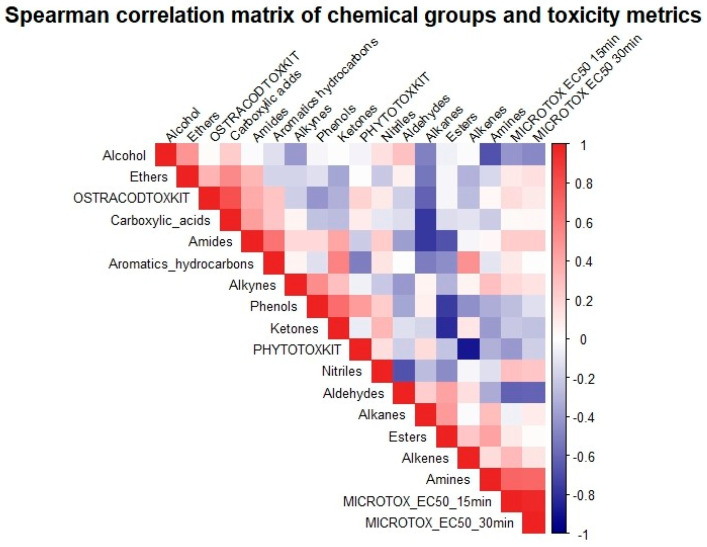
Spearman correlation heatmap for chemical groups and ecotoxicological endpoints. Cells are labeled with r_s_ values. Note. Negative values for root inhibition indicate growth stimulation (potential hormetic effect). For EC_50_ endpoints, lower values indicate higher toxicity; therefore, the toxicological interpretation is inverse to the algebraic sign of r_s_.

To bridge the gap between chemical fingerprinting and biological outcomes, a unified Spearman’s rank correlation matrix was performed (Figure 5). While multivariate techniques were considered, Spearman’s rank correlation was selected as a more robust and conservative approach for this dataset, given the sample size (*n* = 10) and the non-normal distribution of some variables. The analysis included 14 chemical groups (aldehydes, alkanes, alkenes, alkynes, alcohols, amides, amines, aromatic hydrocarbons, esters, ethers, phenols, ketones, carboxylic acids, and nitriles) and four toxicity endpoints: EC_50_ after 15 min, EC_50_ after 30 min, inhibition of mean root length, and ostracod mortality. The missing value denoted as “n.v.d.” was treated as no valid data and excluded pairwise from calculations. Two-sided significance was evaluated at *p* < 0.05. Because lower EC_50_ values indicate higher toxicity, negative correlations with EC_50_ correspond to increasing acute toxicity, whereas positive correlations with root inhibition or mortality correspond to increasing toxicity. It should be noted, however, that while Spearman’s rank correlation identifies monotonic relationships between individual chemical groups and biological endpoints, it may not account for complex toxicological interactions or synergistic effects between different chemical constituents present in the samples.

The correlation matrix revealed only a limited number of statistically significant associations, which was expected given the small sample size (*n* = 10; *n* = 9 for EC_50_ after 30 min due to one missing value). Amines showed the strongest significant positive association with Microtox EC_50_ at both exposure times (r_s_ = 0.71, *p* = 0.022 at 15 min; r_s_ = 0.71, *p* = 0.034 at 30 min), indicating that samples richer in amines tended to display higher EC_50_ values and, therefore, lower acute bacterial toxicity. By contrast, alkenes were significantly negatively correlated with root inhibition (r_s_ = −0.68, *p* = 0.032), which quantitatively supports a stimulatory or hormetic trend rather than phytotoxic inhibition.

Several non-significant but notable tendencies were also observed. Aldehydes were negatively related to both EC_50_ endpoints (r_s_ = −0.62 for 15 min; r_s_ = −0.61 for 30 min), suggesting a possible contribution to acute toxicity toward *Aliivibrio fischeri*. Alcohols showed moderate negative correlations with EC_50_ (r_s_ = −0.42 to −0.46), while aromatic hydrocarbons were moderately negatively associated with root elongation (r_s_ = −0.53). In the Ostracoda assay, alkanes exhibited a moderate inverse relationship with mortality (r_s_ = −0.61; *p* = 0.061), whereas amides and nitriles showed moderate positive trends (both r_s_ = 0.39), although these did not reach the adopted significance threshold.

To provide a robust statistical basis for categorizing firefighting runoffs, a dual agglomerative hierarchical clustering analysis (HCA) was performed on both fire scenarios (observations) and chemical functional groups (variables). Prior to analysis, the data were standardized (z-score transformation) to ensure comparability across different concentration ranges. Dissimilarities were calculated using the Euclidean distance metric, and the hierarchy was constructed using Ward’s minimum variance method (Ward.D2).

**Figure 6:**
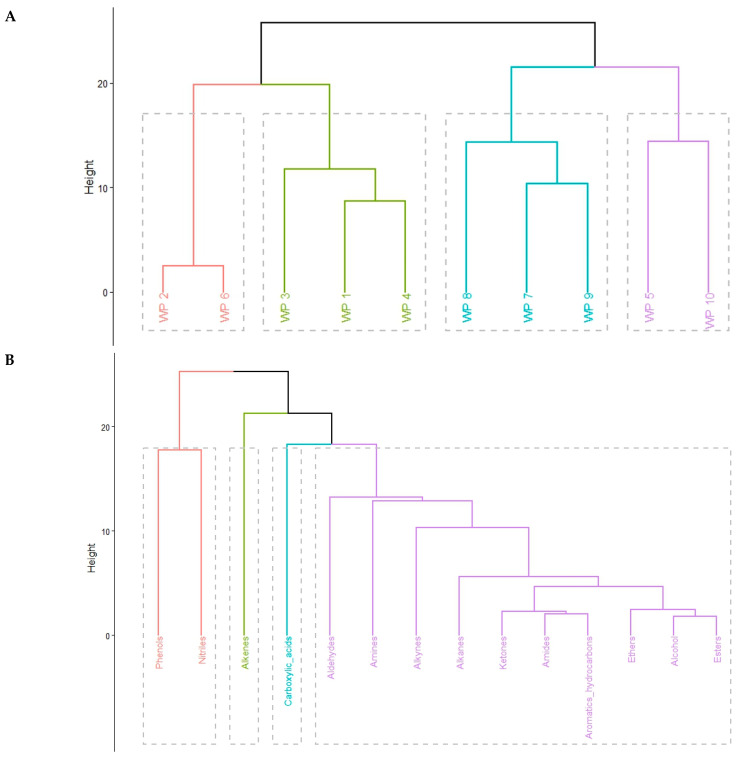
Hierarchical cluster analysis (HCA) of firefighting runoff data: (**A**) dendrogram of fire scenarios identifying similarities between sampling points; (**B**) dendrogram of chemical functional groups illustrating associations between different classes of pollutants.

The resulting HCA of the GC-MS/MS data demonstrates that the chemical “fingerprint” of the effluent is a direct function of the fuel–foam matrix (Figure 6A). The primary bifurcation of the dendrogram into two macro-clusters suggests a fundamental divergence between nitrogen-oxygenated organic loads and lipophilic hydrocarbon-rich matrices. The first cluster (red) shows a high degree of similarity between WP2 (Industrial) and WP6 (Upholstery). Despite their different scales, their chemical redundancy is driven by a high co-variance of alcohols, amides, and esters, as confirmed by the variable clustering in Figure 6B. This association points to a shared thermolysis pathway of polyurethane-based materials and synthetic surfactants. From an environmental management perspective, this implies that industrial and domestic interior fires may discharge nearly identical suites of polar organic compounds, potentially requiring similar localized treatment strategies.

Conversely, the second macro-cluster in Figure 6A highlights the unique risks associated with polymer-dominated fires, specifically WP10 (tires) and WP5 (mixed plastics). The clustering of variables (Figure 6B) further elucidates these signatures; for instance, the close association between phenols and nitriles (red cluster in Figure 6B) serves as a molecular marker for elastomer and synthetic rubber degradation. While WP10 was identified in bioassays as a metabolic outlier (highest Toxi-Chromo LID Score), the HCA confirms that its chemical matrix is fundamentally linked to the thermal breakdown of nitrogen-containing synthetic rubbers. Furthermore, the grouping of alkenes and carboxylic acids in the variable dendrogram suggests coordinated degradation patterns of long-chain polymers. These chemical signatures identified via HCA are not merely a product of the fuel itself but also reflect the complex interaction between the combustible material and the chemical additives used during firefighting interventions.

During fire suppression operations, a variety of extinguishing agents are employed, selected according to the specific nature of the combustible materials and the prevailing incident conditions. Water remains the primary extinguishing medium. However, its efficacy can be insufficient in certain scenarios, particularly regarding the combustion of solids and flammable liquids. Thus, to enhance its performance, additives in the form of aqueous solutions of foam concentrates are widely utilized to generate firefighting foam [28].

From a physicochemical perspective, it is a colloidal system consisting of a mixture of foam solution (a blend of water and concentrate) and air. Depending on the expansion ratio (defined as the ratio of the foam volume to the liquid volume prior to gas induction), firefighting foams are classified as low-expansion (heavy), medium-expansion, and high-expansion (light) foams [28]. According to their chemical composition, foam concentrates are classified into two categories: synthetic and protein-based. The synthetic group is composed of hydrocarbon surfactant-based concentrates (including Class A and wetting agents) and aqueous film-forming foams (AFFFs), which contain both synthetic and fluorinated surfactants. Protein-based agents are formulated using hydrolyzed proteins and are further divided into protein (P), fluoroprotein (FP), and film-forming fluoroprotein (FFFP) types. For the suppression of water-miscible liquids, alcohol-resistant (AR) foams are utilized, as they contain specific polymers that create a protective layer between the fuel and oxygen [29]. The primary extinguishing effect of foam is achieved by separating the surface of the combustible material from the flame. The combustion zone and the fuel are cooled by the foam solution draining from the foam structure. Additionally, the combustion zone is isolated from the air, and fuel evaporation is suppressed. Thermal insulation is also provided, and fire gases are displaced, particularly when rooms are filled or flooded with foam [30]. The fundamental constituents of these concentrates include surfactants, solvents, preservatives, antifreeze agents, corrosion inhibitors, stabilizers, and colorants [31].

Despite their high extinguishing efficiency, firefighting foams impose a significant burden on ecosystems. Previous studies indicate that the environmental impact of foams is primarily associated with the presence of surfactants and solvents. These substances may increase the organic load, measured as Chemical Oxygen Demand (COD) and Biochemical Oxygen Demand (BOD). Furthermore, acute toxic effects may be caused by receiving waters with low dilution, while soil and vegetation can be affected through changes in nutrient availability and surface properties, such as wetting and emulsification [28,30,31].

In the fire scenarios analyzed in this study, the synthetic foaming agent Roteor M-Premium was employed. According to its Safety Data Sheet (SDS), this preparation is a mixture of anionic surfactants (ethoxylated alcohol sulfates, sodium salts-SLES) and co-solvents, including 2-(2-butoxyethoxy)ethanol, ethane-1,2-diol, butan-1-ol, and C_12_–C_14_ alcohol fractions. From a toxicological perspective, the mixture is classified as a skin irritant and as causing serious eye damage. Furthermore, SLES is classified under Aquatic Chronic 3. It should also be noted that the samples were collected at various stages of fire development and suppression operations; this variability could have influenced the dilution levels of both the foam concentrates and the toxic combustion products, which may explain the differences in toxicity results observed between individual samples. These results suggest that despite the presence of irritants and surfactants in the composition of Roteor M-Premium, their concentration in the final fire wastewater did not determine the toxicity level of the samples.

It should be emphasized that the current international literature largely overlooks this aspect, focusing either on the toxicity of the concentrates themselves (under laboratory conditions) or on general water contamination without differentiating between the sources of toxicity. Thus, these results represent a significant novelty, indicating that even when modern, fluorine-free and biodegradable foam concentrates (such as the one used in this study) are applied, the chemistry of the fire itself generates a pollutant load of critical ecotoxicological importance.

The integrated toxicity patterns observed across the ten fire scenarios confirm that firewater runoff induces pronounced and multidimensional toxic effects, capable of triggering both acute lethality and sublethal metabolic disturbances across different trophic levels. These findings are consistent with reports from industrial and wildfire-related events, where runoff transported complex mixtures of combustion residues and firefighting agents into receiving waters [3,13,14,15,32,33]. In our study, the *Aliivibrio fischeri* bioassay proved to be the most sensitive indicator, often reaching near-total bioluminescence inhibition that required significant dilution to determine valid EC_50_ values. This high sensitivity identifies the bacterial component of the ecosystem as the most immediate “target” of acute chemical stress, confirming that the hazard associated with firewater runoff is primarily mixture-driven rather than attributable to individual compounds, a conclusion also reached by Silva et al. [13] and Ré et al. [14] in their investigations of post-fire ash and metal mixtures.

The comparative analysis of trophic sensitivity revealed distinct physiological responses to the same effluents. While samples WP3, WP8, and WP9 induced 100% inhibition in seed germination for *Sinapis alba*, other samples like WP5 and WP6 showed negative inhibition values, reaching as low as −12.25%. This stimulatory effect on root development, or hormesis, likely stems from the presence of diluted nitrogenous compounds or nutrients within the extinguishing agents that act as fertilizers at lower concentrations, masking the underlying toxic potential [3,34]. Karrikins, potent chemical signals derived from smoke and charred organic matter, may promote plant growth. They activate the KAI2/MAX2 signaling pathway, increasing seed sensivity to light and promoting seedling development [34]. However, as demonstrated in studies on biochars, this response is strictly dose- and species-dependent. As concentrations increase, inhibitors begin to dominate over karrikin signals, leading to inhibition of root and shoot growth (Kochanek et al., 2016, Ref. [34]). The observed growth stimulation may mask the actual biochemical stress. Even if a plant’s biomass increases, it may be experiencing severe oxidative stress, as evidenced by a sharp rise in prolonine levels—an amino acid that protects against reactive oxygen species (ROS). Studies on synthetic fire-extinguishing agents, such as Triodol- S, show that even at minimal concentrations, there is a significant decrease in chlorophyll A and B content leading to chlorosis and tissue necrosis after seven-day incubation of *Lemna minor* [35].

The complex nature of phytotoxicity results from the fact that fire effluents are mixtures of natural combustion products, synthetic components of firefighting foams, and other materials present within the fire. This “fire mixture” may exhibit a synergistic effect, where toxins from the foam can impair photosynthesis, induce ROS-mediated oxidative stress and lipid peroxidation in chloroplast membranes, while pyrolytic compounds alter the plant hormonal balance. In the ecosystem scale, both terrestrial and aquatic plants may suffer damage, leading to loss of biotop and filtration functions, which may intensify the transport of pollutants up the food chain.

However, the absolute mortality observed in the Ostracodtoxkit F™ assay for most samples (WP2, WP3, WP6, WP8, WP9, and WP10) underscores the severe risk to benthic invertebrates. These acute toxic responses are consistent with documented severe biological effects following exposure to firewater and ash-contaminated runoff, including genotoxicity in *Chironomus riparius* [15].

The long-term environmental implications of these discharges are further complicated by the interaction between the water column and sediments. Although the present study focused primarily on waterborne toxicity, the extreme responses in ostracods suggest that contaminants bound to particles and ash, which eventually settle in sediments, induce sustained biological effects [14]. As observed by Muñiz González et al. [15], molecular responses in *Chironomus riparius* larvae exposed to wildfire ashes indicate that sediment-bound contaminants may trigger sublethal stress responses even when acute toxicity in the water column decreases due to dilution. Furthermore, the inhibition of primary producers, as seen in the growth suppression of benthic diatoms [36], suggests that the ecological balance may be disrupted far beyond the immediate post-fire period.

Efforts to mitigate this toxicity through biological processes present a significant trade-off. Silva et al. [13] demonstrated that filtration by the freshwater clam *Corbicula fluminea* could reduce the toxicity of ash-loaded runoff, yet this occurred at the cost of increased stress and mortality for the clams themselves. Our chemical profiling, which identified high concentrations of phenols (up to 22%) and nitriles (15.9% in tire-related WP10), suggests that the chemical load in real-world industrial fires would likely exceed the remediation capacity of such invasive species, turning biological filtration into a source of secondary ecological collapse rather than a sustainable strategy.

Ultimately, the results of this study underscore the need for integrated, effect-based approaches in assessing environmental risks. The molecular complexity identified, characterized by nitriles, esters (reaching 41% in WP9), and phenols, poses a direct threat to public health through the potential contamination of drinking water reservoirs. Phenolic compounds, acting as neurotoxins and endocrine disruptors, may lead to the formation of highly toxic chlorinated by-products during standard water disinfection. Evidence from major incidents like the Grenfell Tower fire demonstrates that fire-related contamination can result in concentrations of hazardous substances, such as PAHs and dioxins, far exceeding urban soil reference levels [4]. The identification of these “chemical fingerprints” should, therefore, be treated not only as ecological hazards but as high-risk indicators for human chemical safety, necessitating rigorous and prolonged post-fire monitoring of hydrological catchments within the “One Health” framework.

Future studies may focus on developing quantitative methodologies, such as targeted MRM or SIM modes in GC-MS/MS, to precisely determine the concentrations of the identified organic pollutants (e.g., esters, alcohols, phenols) in firefighting runoff. This will enable robust environmental risk assessments by integrating quantitative chemical data with toxicity outcomes from biotests like Microtox, Ostracodtoxkit, and Phytotoxkit. Such advances will strengthen the correlation between contaminant levels and observed ecotoxicological effects, allowing for the development of mitigation strategies for post-fire water contamination.

## 4. Materials and Methods

### 4.1. Fire Incident Characteristics and Sampling Sites

Extinguishing water runoff samples (WP 1–WP 10) were collected directly from the sites of 10 distinct fire incidents in Poland between May and October 2025. The incidents encompassed a wide range of fuel types and fire scales, ranging from small-scale municipal waste fires to large-scale industrial warehouse blazes. Due to the emergency conditions at fire scenes, samples were collected as grab samples without the possibility of in situ measurements of physicochemical parameters. Detailed characteristics of the fire incidents and the corresponding sampling conditions are summarized in Table 3. Immediately after collection, the samples were transported to the laboratory in portable coolers at a temperature of approximately 4 °C. Upon arrival, they were stored under refrigerated conditions (4 °C) until analysis to minimize chemical and biological degradation. Sample analyzes were performed within the first 24 h after their delivery to the laboratory. Regarding firefighting agents, when foaming agents were used (e.g., during the industrial fires WP3 and WP6), the fire services primarily employed a standard synthetic foaming agent, Roteor M Premium, to suppress the high-intensity blazes.

### 4.2. Ecotoxicological Analysis

#### 4.2.1. Bioluminescence Inhibition Assay (Microtox^®^)

Acute toxicity was determined by measuring the inhibition of natural bioluminescence in the marine bacterium *Aliivibrio fischeri* (strain NRRL B-11177). This method is highly valued in wastewater assessment for its rapid response and sensitivity to both organic and inorganic pollutants [37,38]. The assay reagent, consisting of lyophilized bacteria, was rehydrated with Microtox Re-constitution Solution and used within 3 h. Samples were adjusted for salinity (Osmotic Adjusting Solution 22% NaCl) and incubated at a precise temperature of 15 ± 0.5 °C using the Microtox^®^ M500 analyzer. (ModernWater Ltd., York, UK). Bioluminescence intensity was recorded after 15 and 30 min of exposure, and toxicity was expressed as the percentage of light inhibition compared to a non-toxic aqueous control. For the analyzed samples, EC_50_ values were calculated when possible.

#### 4.2.2. Bacterial Enzyme De Novo Synthesis Assay (Toxi-ChromoTest™)

The Toxi-ChromoTest™ (EBPI, Burlington, ON, Canada) was conducted as a rapid colorimetric bioassay to detect the inhibition of the de novo synthesis of β-galactosidase in a mutant strain of *Escherichia coli* (K12 OR85). This strain features a highly permeable cell wall, increasing its vulnerability to toxic stress. The test principle focuses on the ability of toxicants to interfere with metabolic biosynthesis. Stressed, lyophilized bacteria were rehydrated in a recovery cocktail and exposed to serial dilutions of the fire runoff in 96-well microplates for a 90 min incubation period at 37 °C. Subsequently, a chromogenic substrate (p-nitrophenyl-β-D-galactopyranoside) was added, and the plates were incubated for an additional 30 min at 37 °C. A quantitative colorimetric measurement was performed at 615 nm using a microplate reader (BioTek Epoch Microplate Spectrophotometer, Agilent, Santa Clara, CA, USA) equipped with Agilent BioTek ver. Gen5 data analysis software (Agilent, Santa Clara, CA, USA). The absence or reduction in blue color development in the wells indicated enzyme synthesis inhibition, reflecting the toxic potential of the sample.

#### 4.2.3. Seed Germination and Root Growth Assay (Phytotoxkit F™)

Phytotoxicity was assessed using the Phytotoxkit F™ assay, strictly adhering to the ISO 18763 standard for higher plants. The test utilized seeds of *Sinapis alba* (white mustard), a species recognized for its rapid germination and high sensitivity to a wide range of contaminants. Transparent test plates were prepared by moistening with 45 mL of either distilled water (control) or the firefighting runoff sample. The plated were then covered with a black filter paper, and ten calibrated seeds were evenly distributed near the middle ridge of each plate. The plates were incubated vertically at 25 ± 1 °C for 72 h in complete darkness. Following incubation, the number of germinated seeds was recorded, and primary root lengths were measured using open-source ImageJ software ver. 1.54s (NIH, Bethesda, MD, USA) for digital analysis. Results were reported as a percentage of growth inhibition relative to the control group.

#### 4.2.4. Direct-Contact Assay (Ostracodtoxkit F™)

To evaluate the toxicity of pollutants adsorbed on solid particles and at the sediment–water interface, the Ostracodtoxkit F™ bioassay using the benthic freshwater ostracod *Heterocypris incongruens* was employed. This 6-day test followed the ISO 14371 standard and represents the first standardized “direct-contact” microbiotest for evaluating contaminated matrices. Ostracod cysts were hatched in standard freshwater at 25 °C under continuous illumination for 52 h. Freshly hatched neonates were pre-fed with Spirulina powder for 4 h before being transferred to 6-well microplates. Each well contained 1000 µL of reference sediment, 10 organisms, 2 mL of algal food (*Scenedesmus* sp.), and 2 mL of the firefighting runoff sample. After 6-day incubation at 25 ± 1 °C in total darkness, endpoints for mortality and growth inhibition were recorded. Growth inhibition was calculated by comparing the mean shell length of surviving organisms in test samples to those in the control group.

### 4.3. Chemical Analysis

The GC-MS/MS analysis was aimed at identifying a wide range of organic compounds potentially present in the extinguishing water. The screening focused on the following chemical groups: aldehydes, alkanes, alkenes, alkynes, alcohols, amides, amines, aromatic hydrocarbons, esters, ethers, phenols, ketones, carboxylic acids, and nitriles.

#### 4.3.1. Reagents and Standards

High-purity chromatographic grade dichloromethane (DCM, >99.9%) and methanol (99.9%) were sourced from Merck KGaA (Darmstadt, Germany). For the identification of analytes and calculation of retention indices (RIs), a certified reference material containing a mixture of n-alkanes (C_7_–C_33_) at concentrations of 100–200 µg/mL in hexane was utilized (Restek Corporation, Bellefonte, PA, USA).

#### 4.3.2. Sample Preparation and Extraction Procedure

For analyte isolation, liquid–liquid extraction (LLE) protocol was employed: 1000 mL of each aqueous sample was treated with 20 mL of DCM. The mixture was agitated for 60 min using an orbital shaker (Stuart SI500, TEquipment, NJ, USA) at ambient temperature to ensure optimal phase contact. Following phase separation, the organic fractions were isolated and concentrated to dryness under a gentle stream of nitrogen. The resulting residues were reconstituted in 100 µL of DCM prior to instrumental analysis.

#### 4.3.3. GC-MS/MS Instrumental Parameters

Qualitative chemical profiling was performed using a Shimadzu GCMS-TQ8040 system (Shimadzu Corp., Kyoto, Japan) equipped with a tandem mass spectrometer and an AOC-20ia autosampler. Analyte separation was achieved on a Zebron™ ZB-5MSi capillary column (30 m × 0.25 mm i.d., 0.25 µm film thickness; Phenomenex, Torrance, CA, USA) as described previously [39].

Sample extracts (2 µL) were injected via a splitless injection system maintained at 250 °C and higher pressure at 100 kPa. Ultra-high purity helium (99.999995%, Air Products, Gdansk, Poland) served as the carrier gas, operating at a constant flow rate of 1 mL/min. The oven temperature program was initiated at 40 °C, followed by a linear ramp of 10 °C/min until reaching a final temperature of 290 °C, which was subsequently held for 2 min to ensure complete elution of high-boiling point analytes.

The mass spectrometer (TQ8040, Shimadzu Corp., Kyoto, Japan) was operated in electron ionization (EI) mode at 70 eV. To protect the filament, data acquisition commenced after a 4 min solvent delay, utilizing a scan range of 45–450 *m*/*z*. The ion source and transfer line temperatures were strictly maintained at 220 °C and 300 °C, respectively, with a stable emission current of 150 µA to ensure reproducible fragmentation patterns.

Chromatographic data were processed with GCMS Postrun Analysis ver. 4.45 software (Shimadzu Corp., Kyoto, Japan). Compounds were identified via similarity searches in the mass spectral libraries of the National Institute of Standards and Technology (NIST11 and NIST11s), and Smart Pesticides Database (v1.03; Shimadzu Corp., Kyoto, Japan; 433 pesticides). The Automatic Adjustment of Retention Time (AART) tool within GCMS solution ver. 4.45 software (Shimadzu Corp., Kyoto, Japan) corrected retention time (RT) shifts from column degradation, temperature fluctuations, or complex matrices, enhancing spectral matches to library standards.

A 1 mg/L n-alkane mix (C_7_–C_33_) was injected under GC conditions to derive retention indices from C_14_–C_33_ hydrocarbons, enabling automated RT recalibration and im-proved qualitative accuracy in MS full-scan mode, serving as a reference for retention index calculation and RT alignment. Moreover, MS ion source tuning with perfluorotributyl-amine (PFTBA) preceded analyses, ensuring optimal mass accuracy.

## 5. Conclusions

The chemical and ecotoxicological characterization of firefighting runoff from diverse industrial and municipal incidents demonstrates that these effluents constitute a high-risk, chemically heterogeneous waste stream. This research confirms that the ‘chemical fingerprint’ of the runoff is strictly dictated by the principal fuel, with the identification of specific molecular markers—such as aromatic nitriles (16% in WP10) and high phenolic loads (up to 22% in WP1 and WP8)—serving as diagnostic indicators of the thermal degradation of elastomers and synthetic polymers. The integration of multi-trophic bioassays revealed that these compounds are primary drivers of acute metabolic suppression across different biological levels. A critical finding is the synergistic toxicity of firefighting foams, where alcohols and surfactants act as co-solvents, significantly increasing the bioavailability of hydrophobic xenobiotics and thereby leading to total lethality in aquatic invertebrates and bacteria.

The study further identifies a ‘hormetic trap’ in environmental monitoring; while nitrogenous amides from polyurethane combustion may stimulate plant growth, the same matrix remains lethal to other trophic levels, necessitating a mandatory multi-species approach to avoid underestimating ecological risks. From a public health perspective, the persistence of these molecular groups poses a severe threat to groundwater safety, as they serve as precursors to toxic disinfection by-products in drinking water systems. Given that the majority of tested samples reached Class IV toxicity, these results provide a strong scientific basis for the mandatory retention and specialized treatment of firefighting runoff. These findings shift the assessment of firefighting operations from simple chemical monitoring toward a functional, holistic understanding of the long-term risks to both aquatic biodiversity and human health.

## Figures and Tables

**Table 1:** Acute toxicity of firefighting runoff samples (WP1–WP10) towards the bioluminescent marine bacterium *Aliivibrio fischeri* expressed as EC_50_ values, toxicity units (TUs), and toxicity classification according to Persoone et al. [23].

Sample	EC_50_ After 15 min [%]	TU 15 min	EC_50_ After 30 min [%]	TU 30 min	Persoone Toxicity Class *
WP1	10	10.0	10	10.0	II
WP2 **	8	12.5	6	16.7	III
WP3 **	7	14.3	n.v.d	n.v.d	III
WP4	4	25.0	4	25.0	III
WP5	18	5.6	17	5.9	II
WP6 **	30	3.3	32	3.1	II
WP7	7	14.3	7	14.3	III
WP8	5	20.0	4	25.0	III
WP9 **	8	12.5	8	12.5	III
WP10 **	50	2.0	50	2.0	III

* Toxicity classes: Class I—no acute toxicity (TU < 0.4); Class II—slight acute toxicity (0.4 ≤ TU < 1); Class III—acute toxicity (1 ≤ TU < 10); Class IV—high acute toxicity (10 ≤ TU < 100); Class V—very high acute toxicity (TU ≥ 100). Toxic units (TUs) were calculated as TU = 100/EC_50_, according to Persoone et al. [23]; ** EC_50_ were calculated for diluted samples, final toxicity classification: class IV; n.v.d.—no valid data.

**Table 2:** Phytotoxicological assessment and toxicity classification of firefighting runoff (WP1–WP10) based on root growth inhibition and seed germination parameters.

Sample	Inhibition of Mean Root Length [%]	Inhibition of Longest Root [%]	Mean Seed Germination	Persoone Toxicity Class *
WP1	26.83	32.42	9	II
WP2	75.59	79.56	2	IV
WP3	100	100	0	IV
WP4	25.49	28.86	8	II
WP5	2.04	−4.57	8	I
WP6	−12.25	1.07	8	I
WP7	34.16	27.95	9	II
WP8	100	100	0	IV
WP9	100	100	0	IV
WP10	33.14	33.34	10	II

* Toxicity classes: Class I—no acute toxicity (TU < 0.4); Class II—slight acute toxicity (0.4 ≤ TU < 1); Class III—acute toxicity (1 ≤ TU < 10); Class IV—high acute toxicity (10 ≤ TU < 100); Class V—very high acute toxicity (TU ≥ 100). Toxic units (TUs) were calculated as TU = 100/EC_50_, according to Persoone et al. [23].

**Table 3:** Characteristics of fire incidents and firefighting runoff sampling parameters.

Sample ID	Incident Location	Incident Date	Principal Fuel/Materials Burned	Fire Area (m^2^)	Extinguishing Water Used (m^3^)	Foam Concentrate Used (m^3^)
WP 1	Kobylnica	14 May 2025	Auto workshop, electronics, paints, oils	2250	290	1.5
WP 2	Buk	4 July 2025	Retail grocery store (commercial building)	1000	200	N/A
WP 3	Warsaw	23 July 2025	Municipal waste collection point	200	100	N/A
WP 4	Szczodrzejewo	25 August 2025	Sawmill/Carpentry (wood, lacquers)	250	200	N/A
WP 5	Szczecin	24 August 2025	Shredded tires and pyrolytic oil	1100	300	1.0
WP 6	Jankowy	24 August 2025	Upholstery warehouse (PUR foam)	2100	500	0.05
WP 7	Zalasewo	1 September 2025	Carpentry workshop (wood products)	150	30	N/A
WP 8	Belzyce	1 September 2025	Municipal waste landfill	3000	1220	5.0
WP 9	Lawki	2 September 2025	Heavy vehicles, tires, forest area	900	170	1.5
WP 10	Poznan	13 October 2025	Tire storage/landfill	450	60	1.3

## Data Availability

The original contributions presented in this study are included in the article. Further inquiries can be directed to the corresponding author.
